# Polydatin Prevents Methylglyoxal-Induced Apoptosis through Reducing Oxidative Stress and Improving Mitochondrial Function in Human Umbilical Vein Endothelial Cells

**DOI:** 10.1155/2017/7180943

**Published:** 2017-09-13

**Authors:** Ningbo Pang, Tangting Chen, Xin Deng, Ni Chen, Rong Li, Meiping Ren, Yongjie Li, Mao Luo, Haiyan Hao, Jianbo Wu, Liqun Wang

**Affiliations:** ^1^Drug Discovery Research Center, Southwest Medical University, 319 Zhongshan Road, Luzhou, Sichuan 646000, China; ^2^Laboratory for Cardiovascular Pharmacology of Department of Pharmacology, The School of Pharmacy, Southwest Medical University, 319 Zhongshan Road, Luzhou, Sichuan 646000, China; ^3^Key Laboratory of Ministry of Education for Medical Electrophysiology and the Institute of Cardiovascular Research, Southwest Medical University, 319 Zhongshan Road, Luzhou, Sichuan 646000, China; ^4^Department of International Medicine, University of Missouri School of Medicine, 5 Hospital Drive, CE344–DC095.00, Columbia, MO 65212, USA

## Abstract

Methylglyoxal (MGO), an active metabolite of glucose, has been reported to induce vascular cell apoptosis in diabetic complication. Polydatin (PD), a small natural compound from *Polygonum cuspidatum*, has a number of biological functions, such as antioxidative, anti-inflammatory, and nephroprotective properties. However, the protective effects of PD on MGO-induced apoptosis in endothelial cells remain to be elucidated. In this study, human umbilical vein endothelial cells (HUVECs) were used to explore the effects of PD on MGO-induced cell apoptosis and the possible mechanism involved. HUVECs were pretreated with PD for 2 h, followed by stimulation with MGO. Then cell apoptosis, reactive oxygen species (ROS) generation, mitochondrial membrane potential (MMP) impairment, mitochondrial morphology alterations, and Akt phosphorylation were assessed. The results demonstrated that PD significantly prevented MGO-induced HUVEC apoptosis. PD pretreatment also significantly inhibited MGO-induced ROS production, MMP impairment, mitochondrial morphology changes, and Akt dephosphorylation. These results and the experiments involving N-acetyl cysteine (antioxidant), Cyclosporin A (mitochondrial protector), and LY294002 (Akt inhibitor) suggest that PD prevents MGO-induced HUVEC apoptosis, at least in part, through inhibiting oxidative stress, maintaining mitochondrial function, and activating Akt pathway. All of these data indicate the potential application of PD for the treatment of diabetic vascular complication.

## 1. Introduction

Endothelial cells that form the inner lining of all blood vessels play an import role in various aspects of vascular biology, including blood clotting, barrier function, vasoconstriction, and vasodilation [1]. Endothelial cell dysfunction and/or apoptosis has been considered as one of the most critical events in several diseases, including diabetes mellitus [2, 3], atherosclerosis [4, 5], and thrombosis [6, 7]. Therefore, agents that protect the endothelial cell from dysfunction and/or apoptosis are thought to reduce the incidence of cardiovascular disease.

Methylglyoxal (MGO) is a highly reactive dicarbonyl metabolite generated endogenously from the nonenzymatic degradation of the glycolytic intermediates, glyceraldehyde-3-phosphate and dihydroxyacetone phosphate [8]. Under physiological conditions, MGO is reduced to D-lactate by glyoxalase I, which prevents MGO accumulation [9]. However, in diabetes, the production of MGO is increased due to reduced glyoxalase I activity, resulting to MGO accumulation [10, 11]. MGO reacts with arginine or lysine residues of proteins, leading to the formation of advanced glycation end products (AGEs) and the subsequent activation of the receptor of AGEs (RAGE), which then initiates the vascular complications of diabetes [12, 13]. However, MGO can also directly impair cell functions independent of the AGE-RAGE pathway. Several reports have shown that MGO can induce endothelial cell apoptosis [14–17], mainly through reactive oxygen species (ROS) generation [14–17] and mitochondrial membrane potential (MMP) impairment [15, 17, 18], although the molecular mechanism underlying this process is not yet fully understood.

**Figure 1:**
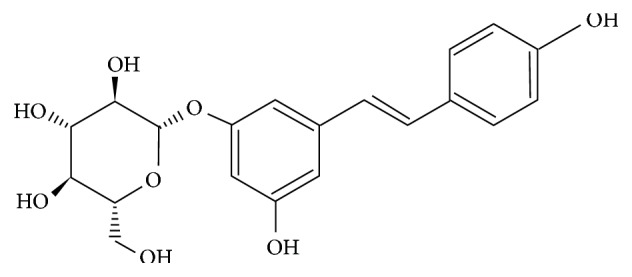
Chemical structure of PD.

Polydatin (PD) (3,4′,5-trihydroxystibene-3-*β*-mono-D-glucoside) (Figure 1) is one of the major active compounds originally extracted from the root and rhizome of *Polygonum cuspidatum* Sieb.et Zucc, a traditional Chinese herbal medicine. It is also detected in red wine, grape hop cones, peanuts, cocoa-containing products, and many daily diets [19]. Previous studies have indicated that PD has a number of biological activities, such as protective effects against shock [20–22] and ischemia/reperfusion damage [23, 24], inhibiting platelet aggregation [25], reducing lipid peroxidation [26], and so on. It has also been demonstrated that PD acts as an antioxidant agent [24, 27] or a mitochondria protector [21, 22] to prevent severe disease. However, the effects of PD on MGO-induced endothelial cell apoptosis have not been reported.

Therefore, the present study aimed to observe the effects of PD on MGO-induced endothelial cell apoptosis. We hypothesized that PD prevents MGO-induced human umbilical vein endothelial cell (HUVEC) apoptosis through reducing oxidative stress and inhibiting mitochondrial dysfunction.

## 2. Materials and Methods

### 2.1. Chemicals and Reagents

PD was from Chengdu Push Bio-Technology (Chengdu, Sichuan, China). PD was dissolved in dimethyl sulfoxide (DMSO) at a stock solution of 10 mM and directly diluted in medium to appropriate concentrations prior to the experiments. MGO was from Sigma (St. Louis, MO, USA). Primary human umbilical vein endothelial cells (HUVECs) and Medium 200 were from Cascade Biologics (Portland, OR, USA). Terminal deoxynucleotidyl transferase dUTP nick end labeling (TUNEL) kits, total superoxide dismutase (SOD) assay kits, catalase (CAT) assay kits, glutathione peroxidase (GSH-Px) assay kits, 2′,7′-dichlorofluorescein diacetate (DCFH-DA), and 5,5′,6,6′-tetrachloro-1,1′,3,3′-tetraethylbenzimidazolcarbocyanine iodide (JC-1) were from Beyotime Biotechnology (Shanghai, China). Annexin V-fluorescein isothiocyanate (FITC)/propidium iodide (PI) apoptosis detection kits were from BD Biosciences (San Diego, CA, USA). Antibodies to phosphorylated Akt, total Akt, Bax, Bcl-2, cleaved caspase-3, and GAPDH were from Cell Signaling Technology (Beverly, MA, USA). N-acetyl cysteine (NAC), Akt inhibitor, and LY294002 (LY) were from Beyotime Biotechnology. Cyclosporin A (CsA) was from Gene Operation (Ann Arbor, Michigan, USA). All other chemicals and reagents were from Sigma unless otherwise indicated.

### 2.2. Cell Culture

HUVECs were grown in Medium 200 containing low-serum growth supplement (LSGS). Cells used were passaged 3–7 times.

### 2.3. Apoptosis Assay

HUVECs were pretreated with PD (0, 25, 50, and 100 *μ*M) for 2 h, followed by stimulation with MGO (200 *μ*M). In some experiments, HUVECs were pretreated with NAC (10 mM), CsA (1 *μ*M), LY (50 *μ*M), or vehicle control for 2 h before MGO (200 *μ*M) treatment. After 24 h incubation at 37°C in a humidified chamber with 5% CO_2_, cells were trypsinized, resuspended in calcium-enriched buffer, stained with Annexin V-FITC and PI for 15 min, and then analyzed by flow cytometry (Guava easyCyte 8HT, Millipore, Boston, MA, USA). Data were calculated with the cell Quest Software. Cell apoptosis was also determined with a TUNEL kit according to the manufacturer's instructions, and all of the nuclei were stained blue with 4′,6-diamino-2-phenylindole (DAPI). The numbers of TUNEL-positive HUVECs and total cells were counted, and apoptosis was evaluated by the ratio of positively stained cells to the total number of HUVECs.

### 2.4. Measurement of Intracellular ROS

HUVECs were pretreated with PD (0, 25, 50, and 100 *μ*M) for 2 h, followed by stimulation with MGO (200 *μ*M) for 1 h. Then cells were washed with Medium 200 and subsequently incubated with DCFH-DA (10 *μ*M) for 30 min at 37°C. After incubation, the fluorescence of the cells was measured using a Spectra Max M5 microplate reader (Molecular Devices, Sunnyvale, CA, USA) (485/530 nm). The fluorescence images were also captured with an EVOS inverted microscope (AMG, Mill Creek, WA, USA).

### 2.5. Measurement of Intracellular SOD, CAT, and GSH-Px Level

HUVECs were plated on 6 cm wells and grown to confluence. Then cells were exposed to serum-free medium for 12 h and treated with PD (0, 25, 50, and 100 *μ*M) for 2 h, followed by stimulation with MGO (200 *μ*M) for 1 h. Cell lysates were prepared and the protein concentrations were determined using a BCA protein assay. Then level of SOD, CAT, and GSH-Px was measured by the respective kits according to the manufacturer's introductions.

### 2.6. Measurement of MMP

JC-1 staining was used to determine MMP as described. HUVECs were pretreated with PD (0, 25, 50, and 100 *μ*M) for 2 h, followed by stimulation with MGO (200 *μ*M) for 1 h. Then cells were incubated with JC-1 (10 *μ*g/mL) for 20 min at 37°C and washed with PBS for 3 times. The fluorescence intensity of JC-1 monomers (490/530 nm) and JC-1 aggregates (525/590 nm) was measured using a Spectra Max M5 microplate reader. The ratio of monomeric to aggregated JC-1 fluorescence intensity was calculated to evaluate changes in MMP. The fluorescence images of JC-1 monomers and JC-1 aggregates were also monitored with an EVOS inverted microscope.

### 2.7. Morphological Observation of Mitochondria

The morphological changes of HUVEC mitochondria were observed with transmission electron microscopy (TEM). HUVECs were fixed with 2.5% glutaraldehyde, stained with cacodylate-buffered osmium tetroxide, and embedded in epoxy resin. Sections were prepared and examined using an electron microscope (Philips CM10, Philips, Eindhoven, Netherlands).

### 2.8. Immunoblotting

HUVECs (3 × 10^5^/well) were plated on 3.5 cm wells and grown to confluence. Then cells were exposed to serum-free medium for 12 h and treated with PD (0, 25, 50, and 100 *μ*M) for 2 h, followed by stimulation with MGO (200 *μ*M) for 1 h. Cell lysates were prepared as described. Protein samples were separated by SDS-PAGE and transferred onto polyvinylidene fluoride (PVDF) membranes (Bio-Rad Laboratories, Hercules, CA, USA). The membranes were blocked with 5% nonfat dry milk solution for 1 h at room temperature. The blocked membranes were probed with antibodies against Bax (1 : 1000), Bcl-2 (1 : 1000), cleaved casepase-3 (1 : 500), phosphorylated Akt (1 : 1000), total Akt (1 : 1000), and GAPDH (1 : 1000) overnight at 4°C. After washing with phosphate-buffered saline containing 0.05% Tween 20, the membranes were incubated with a horseradish peroxidase-conjugated secondary antibody (Santa Cruz Biotechnology) specific to the primary antibody. After further washes, membranes were treated with enhanced chemiluminescence reagents (Merck Millipore, Watford, UK) and protein signal was imaged using ChemiDoc XRS (Bio-Rad Laboratories). ImageJ was used to measure the density of bands.

### 2.9. Data Analysis

All data were expressed as mean ± standard deviation (SD) of the mean. Results were analyzed by one-way analysis of variance (ANOVA) followed by post hoc comparison. *P* < 0.05 was considered to be significantly different.

## 3. Results

### 3.1. PD Prevents MGO-Induced HUVEC Apoptosis

**Figure 2:**
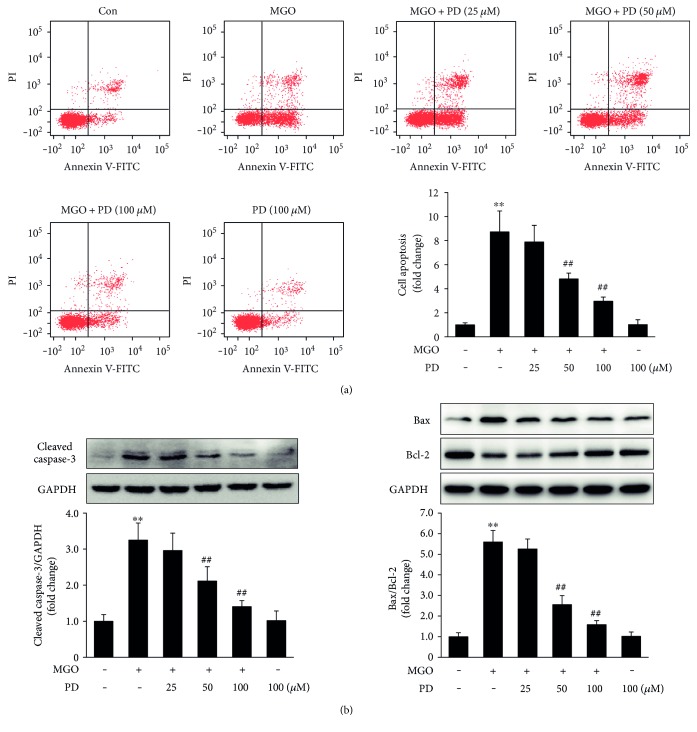
PD prevents MGO-induced HUVEC apoptosis. HUVECs were pretreated with PD for 2 h, followed by stimulation with MGO (200 *μ*M) for 24 h. (a) Cells were trypsinized and stained with Annexin V-FITC and PI for 15 min. Then apoptosis was analyzed by flow cytometry. Representative images of cell population distribution are shown, and quantitative assessment of 3 independent cell apoptosis experiments was performed. Data shown are mean ± SD and are expressed as fold changes. ^∗∗^*P* < 0.01 versus Con; ^##^*P* < 0.01 versus MGO. (b) Expression of cleaved caspase-3, Bax, and Bcl-2 was analyzed by Western blotting. Representative images of 3 independent experiments and densitometric analysis of the levels of cleaved caspase-3 normalized to GAPDH and Bax normalized to Bcl-2 are shown. Data shown are mean ± SD and are presented as fold changes. ^∗∗^*P* < 0.01 versus Con; ^##^*P* < 0.01 versus MGO.

Previous studies have reported that MGO can induce apoptosis in human vascular cells [14–17]. In order to investigate the protective effects of PD on MGO-induced endothelial cell apoptosis, HUVECs were pretreated with PD (0, 25, 50, and 100 *μ*M) for 2 h, followed by stimulation with MGO (200 *μ*M) for 24 h. Apoptotic cells were detected by flow cytometry based on Annexin V-FITC/PI double staining. MGO significantly increased the number of apoptotic cells compared to vehicle control, and the enhanced apoptosis were significantly inhibited by PD at 50 and 100 *μ*M (Figure 2(a)). TUNEL staining also showed similar results (in Supplementary Figure 1 available online at https://doi.org/10.1155/2017/7180943).

The activation of caspase-3, hallmark apoptotic execution enzymes, and the expression of proapoptotic protein, Bax, antiapoptotic protein, and Bcl-2 were also examined by Western blotting. The data showed that the expression of cleaved caspase-3 and the ratio of Bax/Bcl-2 significantly increased with MGO stimulation but decreased by PD pretreatment (Figure 2(b)). Taken together, these results indicate that PD prevents MGO-induced HUVEC apoptosis.

### 3.2. PD Decreases MGO-Induced Oxidative Stress

**Figure 3:**
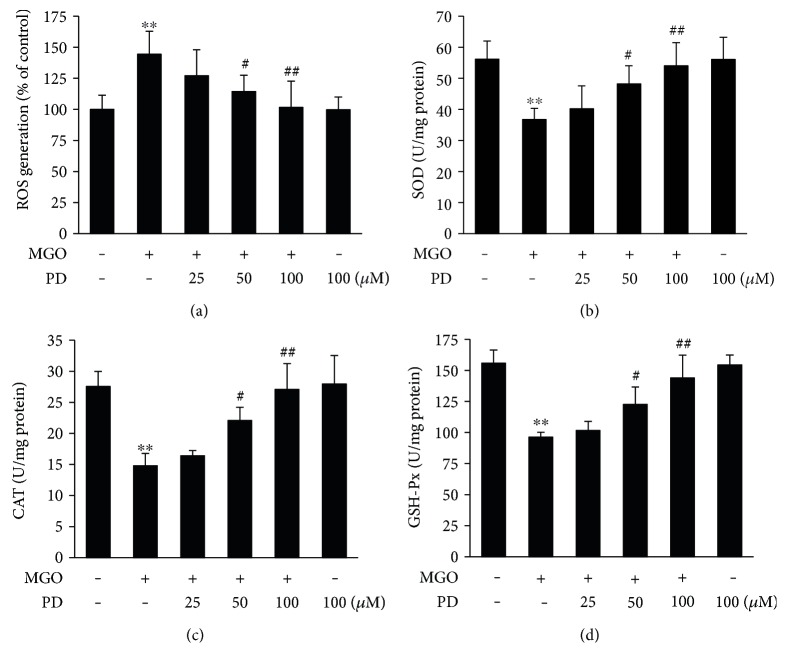
PD decreases MGO-induced oxidative stress. HUVECs were pretreated with PD for 2 h, followed by stimulation with MGO (200 *μ*M) for 1 h. (a) Cells were stained with DCFH-DA, and the fluorescence intensity was measured at 488/525 nm using a microplate reader. Data shown are mean ± SD of 3 independent experiments and are presented as % of control (first bar). ^∗∗^*P* < 0.01 versus Con; ^#^*P* < 0.05 versus MGO; ^##^*P* < 0.01 versus MGO. (b, c, d) The level of SOD, CAT, and GSH-Px was measured by the respective kits according to the manufacturer's introductions. Data shown are mean ± SD of 4 independent experiments. ^∗∗^*P* < 0.01 versus Con; ^#^*P* < 0.05 versus MGO; ^##^*P* < 0.01 versus MGO.

Overproduction of ROS may play an important role in MGO-induced cell apoptosis [14–17]. To determine the effects of PD on intracellular ROS generation, HUVECs were treated with PD (0, 25, 50, and 100 *μ*M) for 2 h and then exposed to MGO for 1 h. Intracellular ROS was significantly increased in cells exposed to MGO. And treatment with PD suppressed ROS production in these cells (Figure 3(a)). Similar results were also shown in fluorescence images (Supplementary Figure 2). The activity of the antioxidant enzymes, such as SOD, CAT, and GSH-Px was also measured. The data showed that the SOD, CAT, and GSH-Px level significantly decreased by MGO stimulation but restored with PD pretreatment (Figures 3(b), 3(c), and 3(d)). These results suggest that PD prevents MGO-induced oxidative stress.

**Figure 4:**
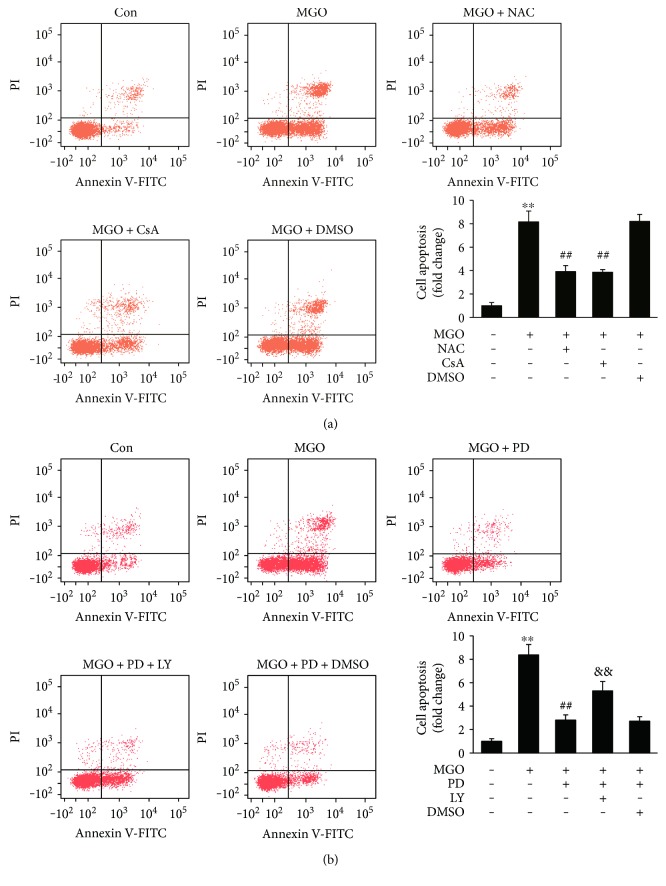
The effects of NAC, CsA, and LY on MGO-induced HUVEC apoptosis. (a) HUVECs were pretreated with NAC (10 mM), CsA (1 *μ*M), or vehicle control for 2 h, followed by stimulation with MGO (200 *μ*M) for 24 h. (b) HUVECs were pretreated with PD (100 *μ*M), PD (100 *μ*M) + LY (50 *μ*M), or vehicle control for 2 h, followed by stimulation with MGO (200 *μ*M) for 24 h. Then cell apoptosis was analyzed by flow cytometry based on Annexin V-FITC/PI double staining. Representative images of cell population distribution are shown, and quantitative assessment of 3 independent experiments was performed. Data shown are mean ± SD and are expressed as fold changes. ^∗∗^*P* < 0.01 versus Con; ^##^*P* < 0.01 versus MGO; ^&&^*P* < 0.01 versus MGO + PD.

To further examine the effects of antioxidant on MGO-induced cell apoptosis, HUVECs were pretreated with NAC (10 mM) for 2 h, followed by stimulation with MGO for 24 h. The results demonstrated that NAC significantly inhibited MGO-induced apoptosis of HUVECs (Figure 4(a) and Supplementary Figure 5(a)). As a whole, these results indicate that PD decreases MGO-induced oxidative stress and the inhibition of oxidative stress is involved in the antiapoptotic effects of PD.

### 3.3. PD Prevents MGO-Induced Mitochondrial Damage

**Figure 5:**
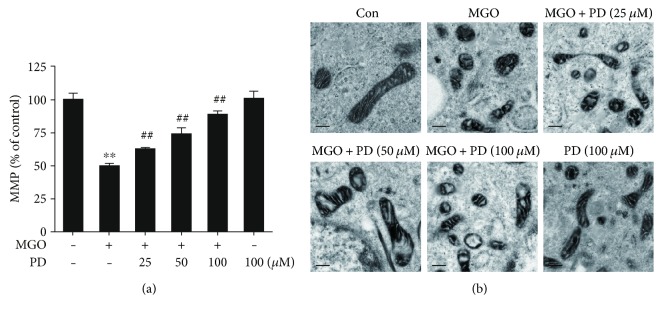
PD inhibits MGO-induced alterations in Akt phosphorylation. HUVECs were pretreated with PD for 2 h, followed by stimulation with MGO (200 *μ*M) for 1 h. Cell lysates were prepared and subjected to Western blotting, to detect the phosphorylation (p) of Akt and total Akt. Representative images of three experiments and densitometric analysis of phosphorylated Akt normalized to total Akt are shown. Data are presented as mean ± SD for 3 independent experiments and are expressed as fold changes. ^∗∗^*P* < 0.01 versus Con; ^##^*P* < 0.01 versus MGO.

Mitochondrial dysfunction has been shown to contribute to the induction of apoptosis. Previous reports have shown that MGO induced the opening of the permeability transition pore (PTP) and significantly decreased the MMP [15, 17, 18]. To explore the effects of PD on MMP, HUVECs were treated with PD (0, 25, 50, and 100 *μ*M) for 2 h and then exposed to MGO for 1 h. MMP was assessed with JC-1 staining. Compared with the control group, MGO significantly decreased the MMP, indicating that the MMP was depolarized (Figure 5(a)). Pretreatment with PD prevented the loss in MMP (Figure 5(a)), which was further supported by the fluorescence images (Supplementary Figure 3). Mitochondrial morphology was also examined using TEM. Cells in control group showed normal mitochondria with preserved membranes and cristae. In contrast, mitochondria appeared swollen and irregularly shaped with disrupted and poorly defined cristae with MGO stimulation. However, PD pretreatment protected the mitochondrial morphological alterations (Figure 5(b)). These data indicate that PD prevents MGO-induced mitochondrial damage.

To identify whether the inhibition of mitochondrial dysfunction was involved in the antiapoptotic effects of PD, HUVECs were pretreated with CsA (an inhibitor of PTP opening) before MGO treatment. MGO-induced cell apoptosis was restored by CsA (Figure 4(a) and Supplementary Figure 5(a)). These results indicate that the protective effects of PD on cell apoptosis are associated with an inhibition of mitochondrial dysfunction.

### 3.4. PD Prevents MGO-Induced Akt Dephosphorylation

**Figure 6:**
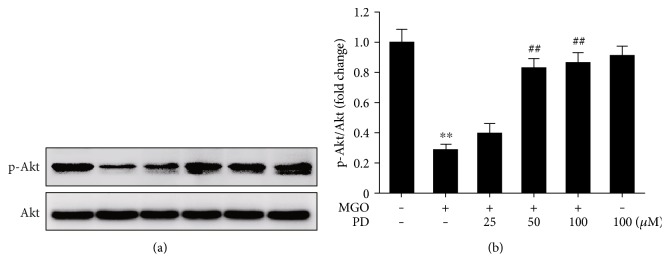
PD prevents MGO-induced mitochondrial damage. HUVECs were pretreated with PD for 2 h, followed by stimulation with MGO (200 *μ*M) for 1 h. (a) The MMP was assessed by JC-1 probe. The fluorescence intensity of JC-1 monomers (490/530 nm) and JC-1 aggregates (525/590 nm) was measured using a microplate reader. The ratio of JC-1 aggregates/JC-1 monomers was calculated. Data shown are mean ± SD for 3 independent experiments and are presented as % of control (first bar). ^∗∗^*P* < 0.01 versus Con; ^##^*P* < 0.01 versus MGO. (b) Ultrastructural alterations of mitochondria were detected by TEM. Representative images of 3 independent experiments are shown. Distance bars: 250 nm.

MGO-induced decrease of Akt phosphorylation has been reported in association with cell apoptosis [17, 28]. Our findings displayed that MGO reduced the phosphorylation as early as in 30 min and the phosphorylation of Akt declined to the nadir in 60 min (Supplementary Figure 4). To explore the effects of PD on Akt phosphorylation, HUVECs were pretreated with PD (0, 25, 50, and 100 *μ*M) for 2 h, followed by stimulation with MGO (200 *μ*M) for 1 h. Pretreatment with PD significantly inhibited MGO-induced Akt phosphorylation reduction (Figure 6). To further identify the effects of blockade of Akt on antiapoptotic effects of PD, HUVECs were pretreated with PD (100 *μ*M), PD (100 *μ*M) + LY (50 *μ*M) (Akt pathway inhibitor), or vehicle control for 2 h, followed by stimulation with MGO (200 *μ*M) for 24 h. LY significantly inhibited the protective effects of PD on MGO-induced apoptosis (Figure 4(b) and Supplementary Figure 5(b)). Taken together, these results indicate that the protective effects of PD against MGO-induced cell apoptosis are partly through re-acting Akt pathway.

## 4. Discussion

It is well known that the concentration of MGO is significantly increased in the plasma of diabetic patients [10] and in vascular endothelial cells exposed to high-glucose media [29]. The abnormal accumulation of MGO has been implicated in causing dysfunction in various tissues and organs [30]. In the present study, we investigated the effects of PD on MGO-induced HUVEC apoptosis *in vitro* and the possible mechanism involved. Our findings suggest that PD prevents MGO-induced HUVEC apoptosis through decreasing oxidative stress, inhibiting mitochondrial damage, and preserving Akt phosphorylation.

MGO has been shown to induce apoptosis in several cell types [15–17, 31–33], including endothelial cells. Annexin V-FITC/PI double staining and TUNEL assay in our study also clearly showed that MGO (200 *μ*M) treatment for 24 h significantly increased HUVEC apoptosis and that PD prevented this injury in a dose-dependent manner. In agreement with these observations, pretreatment with PD prevented the activation of caspase-3 and the increase in Bax/Bcl-2 ratio, thus confirming the cytoprotective properties of PD against MGO-induced apoptosis. These results were consistent with previous studies which demonstrated that PD prevented cell apoptosis against burn [34], shock [35], and ischemia/reperfusion injury [24, 36].

Previous studies [14–17] and our present data showed that oxidative stress significantly increased in endothelial cells in response to MGO treatment. There are several ways by which MGO could increase intracellular ROS. These include generation of superoxide anion and hydrogen peroxide during MGO-induced glycation reaction, consumption of the glutathione content during MGO metabolism, and inactivation of enzymes which can scavenge ROS such as SOD, GSH-Px, and glutathione transferases [37–39]. The present results clearly showed that pretreatment with PD significantly inhibited MGO-induced ROS generation and increased SOD, CAT, and GSH-Px activity in HUVECs. Previous studies demonstrated that ROS production from vessel tissue is a main course leading to endothelial cell injury and as an important upstream signal molecule in MGO-induced apoptosis [14–17]. Consistent with these results, our data also showed that preincubated with antioxidant NAC significantly inhibited MGO-induced apoptosis. Thus, present findings strongly support the hypothesis that PD inhibits MGO-induced apoptotic biochemical changes by blocking ROS formation.

It was previously reported that MGO-induced apoptosis in endothelial cells is also associated with mitochondrial dysfunction. Consistent with previous reports [15, 17, 18], we confirmed in the present study that MGO significantly decreased MMP and altered mitochondrial morphology. Our results also indicated that treatment with PD dose-dependently inhibited the loss in MMP and mitochondrial morphology changes. These results are in agreement with previous reports showing PD as a new mitochondria protector against sepsis [40], shock [20, 21, 35, 41], and ischemia/reperfusion injury [23, 42]. Previous studies have demonstrated that mitochondrial PTP opening is regulated by the activities of Bcl-2 family proteins, including proapoptotic and antiapoptotic proteins, for initiating apoptosis [43]. It is well established that an increase in Bax/Bcl-2 ratio is sufficient to induce mitochondrial PTP opening and to promote cell apoptosis by activating mitochondrial apoptotic signaling pathway [15, 43]. These reports were consistent with our experiments involving the activation of caspase-3 and the increase in Bax/Bcl-2 ratio. Taken together, the results in the present study indicate that the mechanism under protective effects of PD on mitochondria function may be associated with inhibition of mitochondrial PTP opening. Moreover, our experiments involving the inhibitor of PTP opening demonstrated that CsA inhibited MGO-induced apoptosis. Previous studies [15, 17, 18] have also showed that prevention of MMP collapse attenuates MGO-induced cell apoptosis. Therefore, these results suggest that PD prevents MGO-induced apoptosis through protective effects on mitochondrial function.

Akt is a critical component in the phosphatidylinositol 3-kinase (PI3K) pathway that plays a pivotal role in regulating survival and apoptosis of endothelial cells [44]. The antiapoptotic effects of Akt may be associated with regulating the expression of various proteins that are involved in cell death pathways, including Bad, Forkhead, MDM2, and NF-*κ*B [45]. The inhibition of Akt phosphorylation has been reported involving in MGO-induced cell apoptosis [17, 28]. Consistent with previous studies, our data also showed that MGO significantly decreased phosphorylation of Akt, while pretreatment with PD dose-dependently increased the activation of Akt and inhibited MGO-induced apoptosis. In agreement with our results, previous reports have indicated that PD exhibited protective effects against high-fat diet-induced liver damage and ischemia/reperfusion-induced renal injury through upregulating the phosphorylation of Akt [36, 46]. In the present study, the results also demonstrated that LY294002, the PI3K/Akt pathway inhibitor, significantly inhibited the protective effects of PD on cell apoptosis. These results indicate that the upregulation of Akt phosphorylation may be associated with antiapoptotic effects of PD.

It has been suggested that MGO plays an important role in vascular damage to endothelial cells and in the development of vascular disease. Thus, the ability of PD to prevent MGO-induced oxidative stress and apoptosis in endothelial cells may be protective against development of diabetic complications. Although, few experiments have attempted to uncover the direct target of PD in the present study and previous reports and further investigations are needed to better explore the therapeutic potential of PD.

## 5. Conclusions

In this study, we firstly demonstrated that PD exerts protective effects against MGO-induced apoptosis in HUVECs *in vitro*. Our results also suggest that PD prevents MGO-induced apoptosis, at least in part, via inhibiting oxidative stress, maintaining mitochondrial function, and activating Akt pathway. These novel findings indicate the potential application of PD for prevention and treatment of diabetic vascular complications in the clinical practice.

## Supplementary Material

Supplementary figure 1: PD prevents MGO-induced HUVEC apoptosis.HUVECs were pretreated with PD (0, 25, 50 and 100 µM) for 2 h, followed by stimulation with MGO (200 µM). After 24 h, cell apoptosis was examined by TUNEL assay. Representative images of cell apoptosis are shown. Distance bars, 100 µm. Positive cells were counted and quantitative assessment of quadruplicate cell apoptosis experiments was performed. Data shown are mean ± SD and are expressed as fold changes. ∗∗P < 0.01 versus Con, # # P < 0.01 versus MGO. Supplementary figure 2: PD inhibits MGO-induced ROS generation. HUVECs were pretreated with PD (0, 25, 50 and 100 µM) for 2 h, followed by stimulation with MGO (200 µM) for 1 h. Then cells were stained with DCFH-DA. The fluorescence (488/525 nm) was monitored by a fluorescence microscope and representative images of three independent experiments were shown. Distance bar, 100 µm. Supplementary figure 3: PD prevents MGO-induced alterations in MMP. HUVECs were pretreated with PD (0, 25, 50 and 100 µM) for 2 h, followed by stimulation with MGO (200 µM) for 1 h. Then MMP was assessed by JC-1 probe. The fluorescence of JC-1 monomers (Green) (490/530 nm) and JC-1 aggregates (Red) (525/590 nm) was measured using a fluorescence microscope. Representative images of three independent experiments were shown. Distance bars, 100 µm. Supplementary figure 4: MGO time-dependently decreases phosphorylation of Akt. HUVECs were exposed to MGO (200 µM) for 0, 15, 30, 60, 120 and 240 min, as indicated. Cell lysates were prepared and subjected to western blotting, to detect the phosphorylation (p) of Akt and total Akt. Representative images of three experiments and densitometric analysis of phosphorylated Akt normalized to total Akt are shown. Data are presented as mean ± SD for triplicate experiments and expressed as fold changes. #P < 0.05 versus Con, ##P < 0.01 versus Con. Supplementary figure 5: The effects of NAC, CsA and LY on MGO-induced HUVEC apoptosis. (a) HUVECs were pretreated with NAC (10 mM), CsA (1 µM), or vehicle control for 2 h, followed by stimulation with MGO (200 µM) for 24 h. (b) HUVECs were pretreated with PD (100 µM), PD (100 µM) + LY (50 µM), or vehicle control for 2 h, followed by stimulation with MGO (200 µM) for 24 h. Then cell apoptosis was examined by TUNEL assay. Representative images of cell apoptosis are shown. Distance bars, 100 µm. Positive cells were counted and quantitative assessment of quadruplicate cell apoptosis experiments was performed. Data shown are mean ± SD and are expressed as fold changes. ##P < 0.01 versus Con, ##P < 0.01 versus MGO, &&P < 0.01 versus PD.

## References

[1] Urbich C., Dimmeler S. (2004). Endothelial progenitor cells: characterization and role in vascular biology. *Circulation Research*.

[2] van den Oever I. A., Raterman H. G., Nurmohamed M. T., Simsek S. (2010). Endothelial dysfunction, inflammation, and apoptosis in diabetes mellitus. *Mediators of Inflammation*.

[3] Hadi H. A., Suwaidi J. A. (2007). Endothelial dysfunction in diabetes mellitus. *Vascular Health and Risk Management*.

[4] Dimmeler S., Hermann C., Zeiher A. M. (1998). Apoptosis of endothelial cell. Contribution to the pathophysiology of atherosclerosis?. *European Cytokine Network*.

[5] Choy J. C., Granville D. J., Hunt D. W., McManus B. M. (2001). Endothelial cell apoptosis: biochemical characteristics and potential implications for atherosclerosis. *Journal of Molecular and Cellular Cardiology*.

[6] Durand E., Scoazec A., Lafont A. (2004). In vivo induction of endothelial apoptosis leads to vessel thrombosis and endothelial denudation: a clue to the understanding of the mechanism of thrombotic plaque erosion. *Circulation*.

[7] Rajaqopalan S., Somers E. C., Brook R. D. (2004). Endothelial cell apoptosis in systemic lupus erythematosus: a common pathway for abnormal vascular function and thrombosis propensity. *Blood*.

[8] Brenner T., Fleming T., Uhle F. (2014). Methylglyoxal as a new biomarker in patients with septic shock: an observational clinical study. *Critical Care*.

[9] Thornalley P. J. (2003). Glyoxalase I—structure, function and a critical role in the enzymatic defence against glycation. *Biochemical Society Transactions*.

[10] Lapolla A., Flamini R., Dalla Vedova A. (2003). Glyoxal and methylglyoxal levels in diabetic patients: quantitative determination by a new GC/MS method. *Clinical Chemistry and Laboratory Medicine*.

[11] Nemet I., Turk Z., Duvnjak L., Car N., Varga-Defterdarovic L. (2005). Humoral methylglyoxal level reflects glycemic fluctuation. *Clinical Biochemistry*.

[12] Brownlee M. (1994). Glycation and diabetic complications. *Diabetes*.

[13] Giacco F., Brownlee M. (2010). Oxidative stress and diabetic complications. *Circulation Research*.

[14] Phalitakul S., Okada M., Hara Y., Yamawaki H. (2013). Vaspin prevents methylglyoxal-induced apoptosis in human vascular endothelial cells by inhibiting reactive oxygen species generation. *Acta Physiologica*.

[15] Figarola J. L., Singhal J., Rahhar S., Awasthi S., Singhal S. S. (2014). LR-90 prevents methylglyoxal-induced oxidative stress and apoptosis in human endothelial cells. *Apoptosis*.

[16] Do M., Kim S., Seo S. Y., Yeo E. J., Kim S. Y. (2015). *δ*-Tocopherol prevents methylglyoxal-induced apoptosis by reducing ROS generation and inhibiting apoptotic signaling cascades in human umbilical vein endothelial cells. *Food & Function*.

[17] Chu P., Han G., Ahsan A. (2017). Phosphocreatine protects endothelial cells from methylglyoxal induced oxidative stress and apoptosis via the regulation of PI3K/Akt/eNOS and NF-*κ*B pathway. *Vascular Pharmacology*.

[18] Chang T. J., Tseng H. C., Liu M. W., Chang Y. C., Hsieh M. L., Chuang L. M. (2016). Glucagon-like peptide-1 prevents methylglyoxal-induced apoptosis of beta cells through improving mitochondrial function and suppressing prolonged AMPK activation. *Scientific Reports*.

[19] Wu Y., Xue L., Du W. (2015). Polydatin restores endothelium-dependent relaxation in rat aorta rings impaired by high glucose: a novel insight into the PPARβ-NO signaling pathway. *PLoS One*.

[20] Zhao K. S., Jin C., Huang X. (2003). The mechanism of Polydatin in shock treatment. *Clinical Hemorheology and Microcirculation*.

[21] Wang X., Song R., Chen Y., Zhao M., Zhao K. S. (2013). Polydatin-a new mitochondria protector for acute severe hemorrhagic shock treatment. *Expert Opinion on Investigational Drugs*.

[22] Li P., Wang X., Zhao M., Song R., Zhao K. S. (2015). Polydatin protects hepatocytes against mitochondrial injury in acute severe hemorrhagic shock via SIRT1-SOD2 pathway. *Expert Opinion on Therapeutic Targets*.

[23] Miao Q., Wang S., Miao S., Wang J., Xie Y., Yang Q. (2011). Cardioprotective effect of polydatin against ischemia/reperfusion injury: roles of protein kinase C and mito K (ATP) activation. *Phytomedicine*.

[24] Meng Q. H., Liu H. B., Wang J. B. (2016). Polydatin ameliorates renal ischemia/reperfusion injury by decreasing apoptosis and oxidative stress through activating sonic hedgehog signaling pathway. *Food and Chemical Toxicology*.

[25] Liu L. T., Guo G., Wu M., Zhang W. G. (2012). The progress of the research on cardio-vascular effects and acting mechanism of polydatin. *Chinese Journal of Integrative Medicine*.

[26] Du J., Sun L. N., Xing W. W. (2009). Lipid-lowering effects of polydatin from *Polygonum cuspidatum* in hyperlipidemic hamsters. *Phytomedicine*.

[27] Ma Y., Gong X., Mo Y., Wu S. (2016). Polydatin inhibits the oxidative stress-induced proliferation of vascular smooth muscle cells by activating the eNOS/SIRT1 pathway. *International Journal of Molecular Medicine*.

[28] Lv Q., Gu C., Chen C. (2014). Venlafaxine protects methylglyoxal-induced apoptosis in the cultured human brain microvascular endothelial cells. *Neuroscience Letters*.

[29] Mukohda M., Okada M., Hara Y., Yamawaki H. (2012). Exploring mechanisms of diabetes-related macrovascular complications: role of methylglyoxal, a metabolite of glucose on regulation of vascular contractility. *Journal of Pharmacological Sciences*.

[30] Thornalley P. J. (2007). Endogenous alpha-oxoaldehydes and formation of protein and nucleotide advanced glycation endproducts in tissue damage. *Novartis Foundation Symposium*.

[31] Tajes M., Eraso-Pichot A., Rubio-Moscardo F. (2014). Methylglyoxal reduces mitochondrial potential and activates Bax and caspase-3 in neurons: implications for Alzheimer’s disease. *Neuroscience Letters*.

[32] Seo K., Ki S. H., Shin S. M. (2014). Methylglyoxal induces mitochondrial dysfunction and cell death in liver. *Toxicology Research*.

[33] Suh K. S., Choi E. M., Rhee S. Y., Kim Y. S. (2014). Methylglyoxal induces oxidative stress and mitochondrial dysfunction in osteoblastic MC3T3-E1 cells. *Free Radical Research*.

[34] Li T., Cai S., Zeng Z. (2014). Protective effect of polydatin against burn-induced lung injury in rats. *Respiratory Care*.

[35] Zeng Z., Chen Z., Xu S. (2016). Polydatin protecting kidneys against hemorrhagic shock-induced mitochondrial dysfunction via Sirt1 activation and p53 deacetylation. *Oxidative Medicine and Cellular Longevity*.

[36] Liu H. B., Meng Q. H., Huang C., Wang J. B., Liu X. W. (2015). Nephroprotective effects of polydatin against ischemia/reperfusion injury: a role for the PI3K/Akt signal pathway. *Oxidative Medicine and Cellular Longevity*.

[37] Yim H. S., Kang S. O., Hah Y. C., Chock P. B., Yim M. B. (1995). Free radicals generated during the glycation reaction of amino acids by methylglyoxal. *The Journal of Biological Chemistry*.

[38] Choudhary D., Chandra D., Kale R. K. (1997). Influence of methylglyoxal on antioxidant enzymes and oxidative damage. *Toxicology Letters*.

[39] Kang J. H. (2003). Modification and inactivation of human Cn, Zn-superoxide dismutase by methylglyoxal. *Molecules and Cells*.

[40] Gao Y., Zeng Z., Li T. (2015). Polydatin inhibits mitochondrial dysfunction in the renal tubular epithelial cells of a rat model of sepsis-induced acute kidney injury. *Anesthesia and Analgesia*.

[41] Zeng Z., Yang Y., Dai X. (2016). Polydatin ameliorates injury to the small intestine induced by hemorrhagic shock via SIRT3 activation-mediated mitochondrial protection. *Expert Opinion on Therapeutic Targets*.

[42] Gao Y., Chen Y., Lei X. (2016). Neuroprotective effects of polydatin against mitochondrial-dependent apoptosis in the rat cerebral cortex following ischemia/reperfusion injury. *Molecular Medicine Reports*.

[43] Brunelle J. K., Letai A. (2009). Control of mitochondrial apoptosis by the Bcl-2 family. *Journal of Cell Science*.

[44] Osaki M., Oshimura M., Ito H. (2004). PI3K-Akt pathway: its functions and alterations in human cancer. *Apoptosis*.

[45] Matsui T., Rosenzweig A. (2005). Convergent signal transduction pathways controlling cardiomyocytes survival and function: the role of PI3-kinase and Akt. *Journal of Molecular and Cellular Cardiology*.

[46] Zhang Q., Tan Y., Zhang N., Yao F. (2015). Polydatin supplementation ameliorates diet-induced development of insulin resistance and hepatic steatosis in rats. *Molecular Medicine Reports*.

