# Supplementary material for: Polydatin Prevents Methylglyoxal-Induced Apoptosis through Reducing Oxidative Stress and Improving Mitochondrial Function in Human Umbilical Vein Endothelial Cells
Source: Oxid Med Cell Longev. 2017 Sep 13;2017:7180943. doi: 10.1155/2017/7180943 (PMC5615983; doi:10.1155/2017/7180943)
Supplement: Supplementary file 1 — Supplementary figure 1: PD prevents MGO-induced HUVEC apoptosis.HUVECs were pretreated with PD (0, 25, 50 and 100 µM) for 2 h, followed by stimulation with MGO (200 µM). After 24 h, cell apoptosis was examined by TUNEL assay. Representative images of cell apoptosis are shown. Distance bars, 100 µm. Positive cells were counted and quantitative assessment of quadruplicate cell apoptosis experiments was performed. Data shown are mean ± SD and are expressed as fold changes. ∗∗P < 0.01 versus Con, # # P < 0.01 versus MGO. Supplementary figure 2: PD inhibits MGO-induced ROS generation. HUVECs were pretreated with PD (0, 25, 50 and 100 µM) for 2 h, followed by stimulation with MGO (200 µM) for 1 h. Then cells were stained with DCFH-DA. The fluorescence (488/525 nm) was monitored by a fluorescence microscope and representative images of three independent experiments were shown. Distance bar, 100 µm. Supplementary figure 3: PD prevents MGO-induced alterations in MMP. HUVECs were pretreated with PD (0, 25, 50 and 100 µM) for 2 h, followed by stimulation with MGO (200 µM) for 1 h. Then MMP was assessed by JC-1 probe. The fluorescence of JC-1 monomers (Green) (490/530 nm) and JC-1 aggregates (Red) (525/590 nm) was measured using a fluorescence microscope. Representative images of three independent experiments were shown. Distance bars, 100 µm. Supplementary figure 4: MGO time-dependently decreases phosphorylation of Akt. HUVECs were exposed to MGO (200 µM) for 0, 15, 30, 60, 120 and 240 min, as indicated. Cell lysates were prepared and subjected to western blotting, to detect the phosphorylation (p) of Akt and total Akt. Representative images of three experiments and densitometric analysis of phosphorylated Akt normalized to total Akt are shown. Data are presented as mean ± SD for triplicate experiments and expressed as fold changes. #P < 0.05 versus Con, ##P < 0.01 versus Con. Supplementary figure 5: The effects of NAC, CsA and LY on MGO-induced HUVEC apoptosis. (a) HUVECs wer [file 7180943.f1.pdf]

Supplementary Figures:

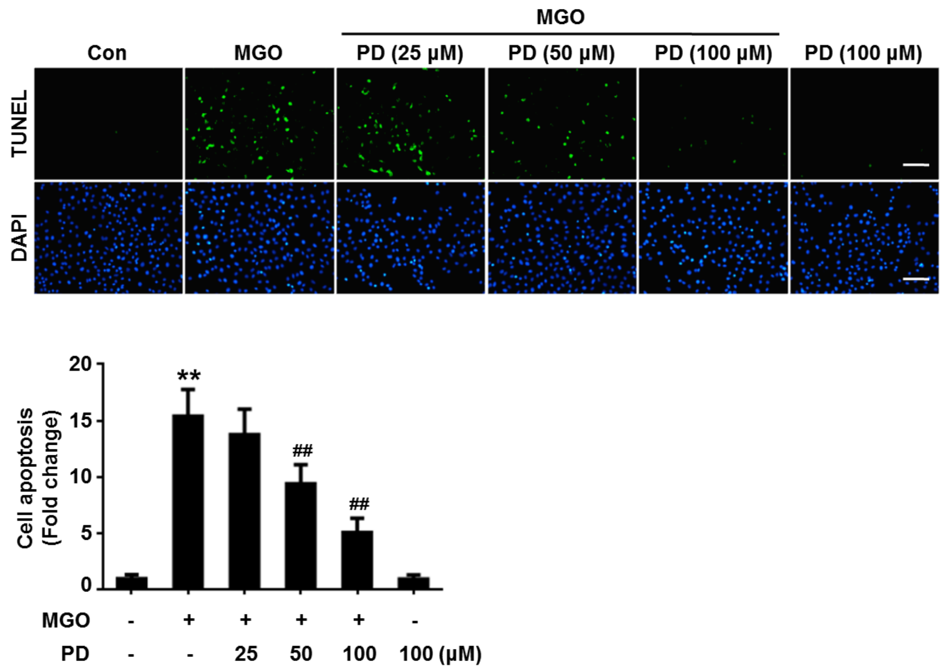

Supplementary figure 1: PD prevents MGO-induced HUVEC apoptosis. HUVECs were pretreated with PD (0, 25, 50 and 100 μM) for 2 h, followed by stimulation with MGO (200 μM). After 24 h, cell apoptosis was examined by TUNEL assay. Representative images of cell apoptosis are shown. Distance bars, 100 μm. Positive cells were counted and quantitative assessment of quadruplicate cell apoptosis experiments was performed. Data shown are mean ± SD and are expressed as fold changes. $^{**}P < 0.01$ versus Con, $^{\#\#}P < 0.01$ versus MGO.

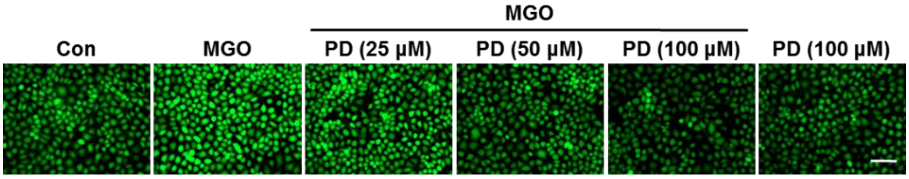

Supplementary figure 2: PD inhibits MGO-induced ROS generation. HUVECs were pretreated with PD (0, 25, 50 and 100 μM) for 2 h, followed by stimulation with MGO (200 μM) for 1 h. Then cells were stained with DCFH-DA. The fluorescence (488/525 nm) was monitored by a fluorescence microscope and representative images of three independent experiments were shown. Distance bar, 100 μm.

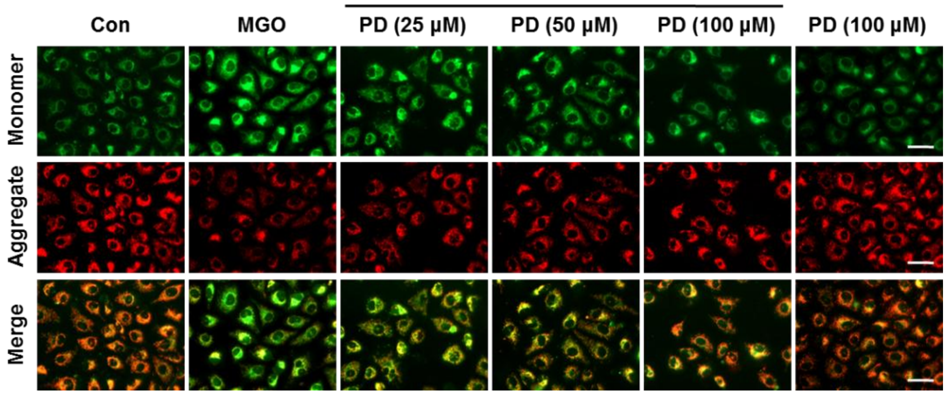

Supplementary figure 3: PD prevents MGO-induced alterations in MMP. HUVECs were pretreated with PD (0, 25, 50 and 100 μM) for 2 h, followed by stimulation with MGO (200 μM) for 1 h. Then MMP was assessed by JC-1 probe. The fluorescence of JC-1 monomers (Green) (490/530 nm) and JC-1 aggregates (Red) (525/590 nm) was measured using a fluorescence microscope. Representative images of three independent experiments were shown. Distance bars, 100 μm.

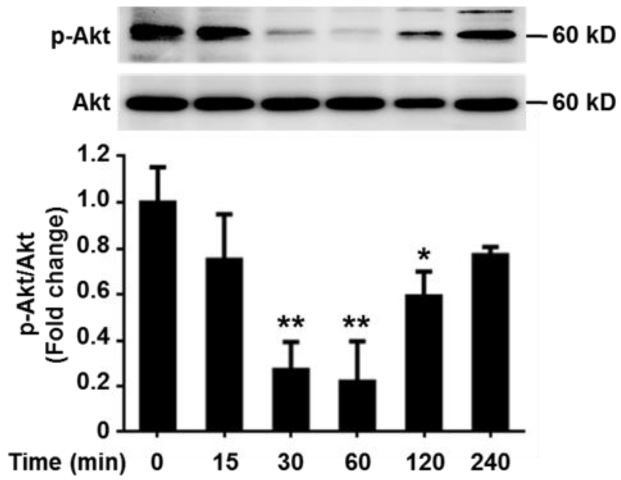

Supplementary figure 4: MGO time-dependently decreases phosphorylation of Akt. HUVECs were exposed to MGO (200 μM) for 0, 15, 30, 60, 120 and 240 min, as indicated. Cell lysates were prepared and subjected to western blotting, to detect the phosphorylation (p) of Akt and total Akt. Representative images of three experiments and densitometric analysis of phosphorylated Akt normalized to total Akt are shown. Data are presented as mean ± SD for triplicate experiments and expressed as fold changes. *P < 0.05 versus Con, **P < 0.01 versus Con.

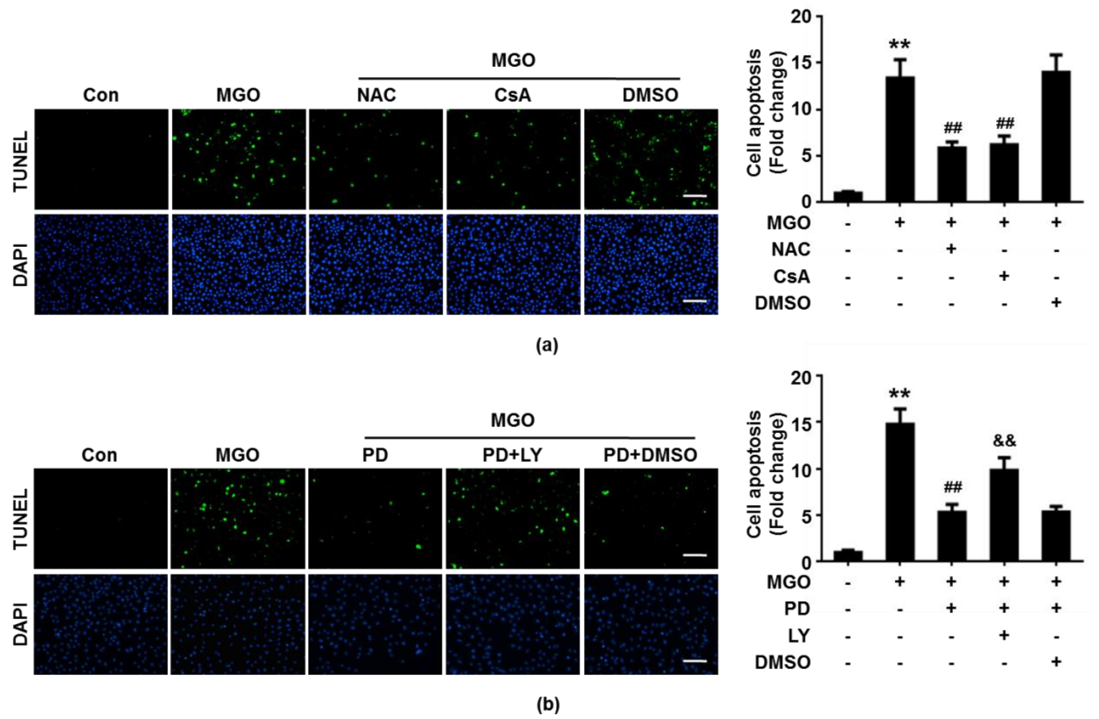

Supplementary figure 5: The effects of NAC, CsA and LY on MGO-induced HUVEC apoptosis. (a) HUVECs were pretreated with NAC (10 mM), CsA (1 μM), or vehicle control for 2 h, followed by stimulation with MGO (200 μM) for 24 h. (b) HUVECs were pretreated with PD (100 μM), PD (100 μM) + LY (50 μM), or vehicle control for 2 h, followed by stimulation with MGO (200 μM) for 24 h. Then cell apoptosis was examined by TUNEL assay. Representative images of cell apoptosis are shown. Distance bars, 100 μm. Positive cells were counted and quantitative assessment of quadruplicate cell apoptosis experiments was performed. Data shown are mean ± SD and are expressed as fold changes. $^{**}P < 0.01$ versus Con, $^{\#\#}P < 0.01$ versus MGO, $^{\&\&}P < 0.01$ versus PD.
